# Psychosocial Determinants of HIV Stigma among Men Who Have Sex with Men in San Francisco, California

**DOI:** 10.3390/ijerph18158031

**Published:** 2021-07-29

**Authors:** Dharma N. Bhatta, Jennifer Hecht, Shelley N. Facente

**Affiliations:** 1 San Francisco AIDS Foundation, San Francisco, CA 94103, USA; dnbhatta@yahoo.com (D.N.B.); jhecht@sfaf.org (J.H.); 2 Division of Epidemiology and Biostatistics, University of California, Berkeley, CA 94720, USA; 3 Facente Consulting, Richmond, CA 94804, USA

**Keywords:** men who have sex with men (MSM), HIV, stigma

## Abstract

Background: Stigma and discrimination are major challenges faced by people living with HIV (PLWH), and stigma continues to be prevalent among PLWH. We conducted a cross-sectional study of 584 men who have sex with men (MSM) living with HIV between July 2018 and December 2020, designed to better understand which demographic and behavioral characteristics of MSM living with HIV in San Francisco, California are associated with experience of stigma, so that programs and initiatives can be tailored appropriately to minimize HIV stigma’s impacts. Methods: This analysis was conducted with data from San Francisco AIDS Foundation (SFAF) encompassing services from multiple different locations in San Francisco. Data about the level of HIV-related stigma experienced were collected through a single question incorporated into programmatic data collection forms at SFAF as part of the client record stored in SFAF’s electronic health record. We performed linear regression to determine the associations between self-reported experiences of HIV stigma and other characteristics among MSM living with HIV. Results: HIV stigma was low overall among MSM living with HIV who are actively engaged in HIV care in San Francisco; however, it was significantly higher for the age groups of 13–29 years (adjusted risk difference (ARD): 0.251, 95% CI: 0.012, 0.489) and 30–49 years (ARD: 0.205, 95% CI: 0.042, 0.367) when compared to the age group of 50 years and older, as well as people who were homeless (ARD: 0.844, 95% CI: 0.120, 1.568), unstably housed (ARD: 0.326, 95% CI: 0.109, 0.543) and/or having mental health concerns (ARD: 0.309, 95% CI: 0.075, 0.544), controlling for race, injection history, and viral load. Conclusions: These findings highlight an opportunity to develop culturally, socially, and racially appropriate interventions to reduce HIV stigma among MSM living with HIV, particularly for younger men and those struggling with housing stability and/or mental health.

## 1. Introduction

HIV transmission among men who have sex with men (MSM) remains a challenge in both developing and developed countries, and combating the HIV burden among this important population is crucial to ending the epidemic [1,2]. Racial and ethnic disparities continue to exist with respect to HIV diagnosis rates. More than two thirds (69%) of new HIV diagnoses were among gay and bisexual men in the United States in the last year [1].

Stigma and discrimination are major challenges faced by people living with HIV (PLWH) [3], and stigma continues to be prevalent among PLWH [4]. PLWH may perceive enacted stigma (discrimination experienced, acts of violence and marginalization), anticipated stigma (negative social perspectives towards HIV, anticipation that a PLWH will experience prejudice and discrimination in the future) and internalized stigma (commendation of negative views, feelings and beliefs of oneself as it relates to one’s HIV-positive status) [4,5]. Stigma can originate from judgmental attitudes and misunderstandings towards social groups that are disproportionately affected by HIV, including MSM, people who inject drugs and ethnic or racial minorities [6,7]. Stigma can exhibit within multiple social domains, including in healthcare systems [8], where stigma can include rejection of care, negative attitudes, embarrassment caused by health worker practices and confidentiality breaches [9,10].

HIV stigma negatively impacts health behaviors, sexual and social relationships, quality of life, mental well-being, and self-esteem of PLWH [11,12]. HIV stigma is associated with poorer physical and mental health outcomes and lower social supports [13], anxiety [14], depression [12], emotional distress [15], shame [16], stress associated with disclosure [17] and suicidal ideation [18]. HIV stigma is a major obstacle to linkage to care and support [19], HIV prevention efforts [20], timely diagnosis, healthcare-seeking behavior [21] and adherence to antiretroviral therapy [22].

PLWH often encounter stigma due to their affiliations with marginalized groups related to gender, sexual orientation, ethnic or racial identity, sex work, poverty, incarceration and/or substance use [23]. Stigma is associated with legal frameworks, societal power structures and interconnecting prejudgments experienced by the marginalized groups of populations disproportionately affected by HIV [24], including MSM, who are doubly exposed to stigma for being both MSM and PLWH [25,26]. Systematic reviews highlight that there remain scarce intervention data on effective stigma reduction interventions for several key populations, including MSM living with HIV in many geographic regions [27,28]. A systematic review of 11 studies emphasized that multilevel intervention strategies are required to mitigate the stigma among MSM living with HIV [28], which remains a major challenge [29] to efforts to control HIV in the United States, in line with the national “Ending the HIV Epidemic” plan, which asserts that all PLWH need timely linkage to care and successful long-term medication adherence and viral suppression [30,31,32]. The objective of this work was to understand the frequency with which MSM living with HIV in San Francisco, California experience HIV-related stigma, and which demographic and behavioral characteristics are associated with experiencing that stigma.

## 2. Methods

### 2.1. Data

This analysis was conducted with data from San Francisco AIDS Foundation (SFAF) encompassing services from multiple different locations in San Francisco. SFAF is a nongovernmental organization serving people to promote health, wellness and social justice (https://www.sfaf.org/ (accessed on 27 July 2021)). Data were collected among those who were diagnosed with HIV, aged 13 and older, willing to self-respond to a question about stigma as part of their regular care and who visited any locations of SFAF to receive sexual health services between July 2018 and December 2020. All people whose data were included in this study provided informed consent for services and related routine programmatic data collection. As this is an internal evaluation of programmatic data, this analysis was not considered human subject research, and no ethical approval was required.

Data about the level of HIV-related stigma experienced were collected through a single question incorporated into programmatic data collection forms at SFAF as part of the client record stored in SFAF’s electronic health record system. At the time of services, clients routinely completed visit forms that included information about their ongoing behaviors, health risks and attitudes or experiences, as well as any updates to demographic information that may have occurred since their prior visit. Additional data (e.g., viral load or other laboratory results) were added to the clinical record by the provider attending to the client during that visit. All entries by clients or providers were dated so that they could be synthesized for this analysis.

### 2.2. Outcome Variable

The level of HIV stigma was determined for the participants who responded “never”, “rarely”, “sometimes”, or “often” to the following question: “In the last 12 months, to what extent have you experienced stigma or discrimination (e.g., avoidance, pity, blame, rejection, verbal abuse or bullying) in relation to your HIV status?” The responses were coded as 0 (never), 1 (rarely), 2 (sometimes) and 3 (often).

### 2.3. Independent Variables

Independent variables included age (13–29 years, 30–49 years, 50 years and above), race (White only; Asian (Asian only or Native Hawaiian or Pacific Islander); Black or African American only; Hispanic or Latinx only; multiple-race (those who reported more than one race category); other race (those who reported a race other than those listed above or reported Middle Eastern, North African, American Indian or Alaska Native race)), injection history for the last 12 months (people who inject drugs (PWID) or no), housing status (homeless: living outdoors or in a vehicle, navigation center or a shelter or having no home; stable housing: living in stable housing, rented or owned; unstable housing: having unstable housing or couch surfing or living in treatment or transitional housing or living in a hotel or staying with a friend), mental health status (yes: have often felt down or depressed or hopeless or have often felt little interest or pleasure in doing things; no) and viral load (detectable: >200 copies/milliliter; undetectable).

### 2.4. Analysis

We calculated the average score and standard deviation of HIV stigma by each of the independent variables. We tested significant differences between HIV stigma and background characteristics using *t*-tests and ANOVA *F*-tests.

We used univariate linear regression to estimate unadjusted associations between HIV stigma and each of the independent variables. We then performed multiple linear regression models to estimate the adjusted association between HIV stigma and background variables adjusting for age, race, injection history, housing, mental health status and viral load. We presented a risk difference (RD) for univariate and adjusted risk difference (ARD) for multiple regression outcomes with 95% confidence intervals.

All the analyses were performed in the R statistical software, version 4.0.5 [33]. Statistical significance was attributed to *p*-values ≤ 0.05. We used listwise deletion in regression analyses (the missing numbers included 1 for housing and 45 for viral load), and the final sample size is included in the table.

## 3. Results

A total of 1690 MSM living with HIV visited SFAF between July 2018 and December 2021. Of those, a total of 584 participants responded to the stigma question. Table 1 provides the breakdown of these participants by background characteristics.

Overall, 352 participants (60.3%) reported never experiencing HIV stigma; 132 (22.6%) responded “rarely”, 86 (14.7%) responded “sometimes”, and 14 (2.4%) reported often experiencing HIV stigma. The average HIV stigma rating was higher among people who were 13–29 years old, of “other” race, had injection history, experienced homelessness, had mental health concerns and/or had a detectable viral load. HIV stigma was significantly different among age groups and by race, housing status and mental health status (Table 2).

Table 3 shows relationships between HIV stigma and background characteristics. In unadjusted regression analyses, age, housing status and mental health status were significantly associated with HIV stigma. After controlling for age, race, injection history, housing, mental health status and viral load, HIV stigma was significantly higher for the age groups of 13–29 years (adjusted risk difference (ARD): 0.251, 95% CI: 0.012, 0.489) and 30–49 years (ARD: 0.205, 95% CI: 0.042, 0.367) when compared to the age group of 50 years and older. The participants who reported being homeless (ARD: 0.844, 95% CI: 0.120, 1.568) and having unstable housing (ARD: 0.326, 95% CI: 0.109, 0.543) experienced significantly higher HIV stigma than those who had stable housing. The participants who reported mental health concerns (ARD: 0.309, 95% CI: 0.075, 0.544) had higher HIV stigma ratings than those who did not.

We performed separate multiple linear regression analyses adjusting for age, race and injection history alone and adjusting for housing, mental health status and viral load alone (Table S1). We noticed that people who reported multiple races had significantly higher HIV stigma than White people after adjusting for age, race and injection history (Table S1) and in unadjusted analysis (Table 3), but after adjusting for all the variables (Table 3), the risk difference for any race categories was not statistically significant compared to the White race, suggestive of possible qualitative interaction between race and another variable.

## 4. Discussion

Our study contributes to the literature by examining the relationship between psychosocial factors and HIV-related stigma among MSM living with HIV in San Francisco, California. Amongst this sample of individuals visiting an HIV service organization in San Francisco (a city known for its long history of HIV services and research), overall reported levels of HIV stigma experienced were quite low. We found that experiences of HIV stigma were positively associated with age, race, homelessness or unstable housing and mental health status.

Our study found that HIV stigma was significantly higher among younger adult MSM living with HIV compared to older MSM. Reports in the literature related to the relationship between age and HIV stigma are inconsistent. Previous studies have found that HIV stigma was not associated with age [34,35] however, a meta-analysis found a negative relationship between stigma and increased age [13], and Batista and Pereira found that older gay and bisexual men (both living with and without HIV) had higher than expected levels of resilience and lower levels of depression and anxiety compared to the general population [36]. A systematic review found that older adults had a significant risk of experiencing HIV-related stigma [37]. One research team suggests that older PLWH have higher stigma because of the dual stigma of having HIV coupled with age discrimination [38,39]. Age does matter when it comes to understanding stigma among PLWH; however, the relationship between age and stigma is complex, and it might depend on several factors (such as route of transmission, personal characteristics, etc.) [39]. The possible reasons for lower levels of stigma among older MSM in San Francisco might include the historical experience of older MSM living throughout the early years of the AIDS epidemic, resilience gained through years of experience, as well as extensive community services provided for older MSM by SFAF and other organizations.

Though the results were not statistically significant, in this study we found that individuals who identified as people of color reported less frequent experiences of HIV stigma compared to those who identified as White when controlling for age, injection history, housing status, mental health status and viral load. Other studies have similarly suggested that HIV stigma is experienced more frequently among White people than among people of color [40,41]. However, it might be expected that people of color would experience more HIV stigma due to having multiple stigmatized identities [42,43]. It has repeatedly been demonstrated that race has substantial impact on health outcomes [44,45,46,47,48]. People of color living with HIV may also face stressors associated with social inequalities, stigma and racism [41,42], and it is necessary to continue to address individual, societal and structural factors which affect experiences of stigma for people of color living with HIV. There may be a number of reasons we see no race-based effect in our multivariate model. First, confidence intervals are wide due to the small number of people in different race categories, and therefore the true effect is quite uncertain. Second, experiences of stigma are complex, and in this study we did not differentiate between the stigma experienced within one’s own community and the stigma experienced from those outside one’s community; this lack of clarity may produce seemingly conflicting results [49].

A systematic review highlighted that MSM may experience multiple layers of stigma based on their HIV status, behavior and sexuality [50]. Another review suggested that trans and gender-nonconforming (TGNC) people living in the US are exposed to a range of social stressors, including stigma, which contribute to adverse health problems [51]. Trans people living with HIV have been found to experience high levels of HIV-related stigma in addition to stigma and discrimination related to their gender identity, both of which are likely to impact their health outcomes [52,53]. In our study, we did not include gender identity in the analysis as our sample was restricted to MSM; however, 15 respondents who were counted as MSM identified their gender identity as genderqueer, transmasculine, or male-identified but gender-nonconforming. Macro- and microinterventions designed to decrease stigma and increase social connectedness are crucial for enhancing the well-being of the TGNC community and may also positively impact people traditionally thought of as MSM [54].

Sexual minorities living with HIV, including MSM, often report higher levels of substance use to manage the toll of HIV-related stigma compared to those who are HIV-negative [55]. Those who inject drugs also frequently experience a combination of HIV-related stigma and drug-related stigma, which is possibly more harmful than experiencing either alone [56]. Though we did not find significantly greater experience of HIV stigma reported among MSM who inject drugs compared to those who did not in our study, the relationship between HIV stigma and substance use is complex. Attention to the intertwined relationship between substance use and stigma is warranted within programs to support MSM living with HIV.

MSM are at increased risk of exclusion from housing services and forced removals from family homes, which makes MSM extremely overrepresented among homeless populations [57]. PLWH suffering homelessness or unstable housing are also impacted by multilayered stigma [58]. Our study found that homelessness and unstable housing was significantly associated with HIV stigma. Lack of adequate, stable and secure housing is a significant barrier to reliable and appropriate access to HIV medical care, access and adherence to antiretroviral therapies and sustained viral suppression among PLWH [54]. Continuous housing assistance and interventions to involve PLWH with housing instability in medical care may help decrease stigma in this population [57,58].

Mental health disorders are also more frequent among MSM living with HIV compared to others [59]. Our analysis found a significant positive association between presence of mental health concerns and HIV stigma. A recent meta-analysis found that HIV stigma is associated with higher rates of depressive symptoms and greater levels of emotional and mental distress among PLWH [12]. Mental health disorders have been associated with HIV stigma among MSM living with HIV and negatively associated with early healthcare-seeking after an HIV diagnosis [59]. Our study was not able to capture the age at onset of mental health concerns and was unable to infer whether mental health concerns existed due to HIV status or other factors. Comprehensive care that supports continued engagement in healthcare and addresses stigma, substance use and mental health is required to better engage MSM in HIV treatment, particularly MSM living with HIV who experience comorbid social and psychological problems.

Finally, the achievement of viral suppression among PLWH is critical to preventing new HIV infections. Our study revealed the association between higher stigma and detectable viral load. Though not statistically significant, these findings were aligned with those of a previous study, which suggested that experiencing HIV-related stigma reduced the likelihood of achieving an undetectable viral load among MSM living with HIV [60]. Another study by Pereira et al. found that having undetectable viral load was an autonomous identity affecting social and interpersonal interactions and therefore potentially impacting experiences of stigma [26]. Especially in the era of Undetectable = Untransmittable, or “U = U” [61], the impact of undetectable viral load will likely have an increasingly positive impact on the reduction of the experience of HIV stigma among MSM and from others outside the MSM community.

### Limitations

The cross-sectional design of this study limits our ability to establish any causal conclusions, and caution should be taken when interpreting study findings. Nonetheless, the positive and negative relationships found here suggest that more interventions are needed to reduce stigma for MSM living with HIV. Our study recruited MSM living with HIV from a single service provider in San Francisco, used convenience sampling techniques and excluded those who were not receiving healthcare, which could limit the generalizability of the findings. However, we would anticipate these factors to lead to an underestimate of the HIV-related stigma experienced by MSM living with HIV in other parts of the United States. HIV status, mental health status, viral load and experiences of stigma were sometimes self-reported and not always verified with laboratory or other supporting data and therefore might be affected by social desirability bias. However, our established trusted care services designed to protect privacy among participants should minimize this effect. We measured HIV-related stigma with a single question which did not attempt to differentiate between the different forms of stigma among MSM living with HIV. This single-question strategy is most suitable for long-term program monitoring and evaluation purposes where clients may be unwilling to respond/fill a series of questionnaires repeatedly across multiple visits. However, as stigma is a complex psychological construct, these findings can only be considered to be exploratory and indicative of a need for more rigorous study using a validated tool to assess stigma more thoroughly. Finally, we were unable to control for socioeconomic status, duration of HIV diagnosis, comorbidities and other possible risk factors in our analysis, which should be considered in the future research. In spite of these limitations, our study describes the context of HIV-related stigma among a racially and socially diverse population with the highest burden of HIV in the United States.

## 5. Conclusions

We designed an analysis to better understand the frequency with which MSM living with HIV in San Francisco, California experience HIV-related stigma and which demographic and behavioral characteristics are associated with experiencing HIV stigma, so that programs and initiatives could be tailored appropriately to minimize the negative effects of HIV stigma in this population. We found that HIV stigma is still prevalent among MSM living with HIV in the United States, especially among MSM under age 50 and those who have housing instability and/or mental health concerns. These findings highlight an opportunity to develop culturally, socially and racially appropriate interventions to reduce HIV stigma among MSM living with HIV, particularly for younger men and those struggling with housing stability and/or mental health. Housing stability is a critical issue in San Francisco which—like many other metropolitan areas that attract MSM with promise of less stigmatization against gay men—has a cost of living that creates great strain and stress for most people living there. The HIV-related stigma we found in this study could be successfully reduced through support groups and other public health interventions that have been demonstrated to effectively reduce the negative impacts of stigma for MSM living with HIV [62,63].

## Figures and Tables

**Table 1:** Individual characteristics.

Variable	*n*	%
**Age in years**		
13–29	96	16.44
30–49	298	51.03
50 and above	190	32.53
**Race**		
Asian	36	6.65
Black or African American	48	8.87
Hispanic or Latinx	142	26.25
Multiple	42	7.76
Other	15	2.77
White	258	47.69
**Injection history**		
No	512	87.67
Yes	72	12.33
**Housing status**		
Stable housing	502	86.11
Homeless	9	1.54
Unstable housing	72	12.35
**Mental health status**		
No	524	89.73
Yes	60	10.27
**Viral load**		
Undetectable	511	94.81
Detectable	28	5.19

**Table 2:** Average HIV stigma score by individual characteristics.

Variable	Mean Score of HIV Stigma (SD)	*p*-Value
**Age in years**		0.003 **
13–29	0.71 (0.87)	
30–49	0.66 (0.86)	
50 and above	0.43 (0.72)	
**Race**		0.031 **
Asian	0.61 (0.80)	
Black or African American	0.60 (0.82)	
Hispanic or Latinx	0.54 (0.80)	
Multiple	0.93 (0.84)	
Other	0.93 (1.03)	
White	0.52 (0.80)	
**Injection history**		0.084 *
No	0.57 (0.81)	
Yes	0.75 (0.92)	
**Housing status**		<0.001 **
Stable housing	0.53 (0.80)	
Homeless	1.33 (1.00)	
Unstable housing	0.92 (0.90)	
**Mental health status**		0.001 *
No	0.56 (0.81)	
Yes	0.92 (0.89)	
**Viral load**		0.186 *
Undetectable	0.58 (0.81)	
Detectable	0.79 (0.96)	

** ANOVA *F*-test, * *t*-test; SD: standard deviation.

**Table 3:** Association between individual characteristics and HIV stigma.

	CRD (95% CI)	*p*-Value	ARD (95% CI)	*p*-Value
**Age in years**				
13–29	0.310 (0.089, 0.531)	**0.006**	0.251 (0.012, 0.489)	**0.039**
30–49	0.254 (0.097, 0.410)	**0.002**	0.205 (0.042, 0.367)	**0.014**
50 and above	Reference		Reference	
**Race**				
Asian	0.097 (−0.193, 0.387)	0.511	−0.009 (−0.301, 0.282)	0.950
Black or African American	0.107 (−0.155, 0.369)	0.421	−0.042 (−0.308, 0.223)	0.753
Hispanic or Latinx	−0.004 (−0.180, 0.172)	0.964	−0.132 (−0.314, 0.050)	0.156
Multiple	0.380 (0.109, 0.650)	**0.006**	0.220 (−0.054, 0.493)	0.115
Other	0.413 (−0.009, 0.834)	0.055	0.131 (−0.297, 0.558)	0.548
White	Reference		Reference	
**Injection history**				
Yes	0.171 (−0.050, 0.392)	0.129	0.071 (−0.149, 0.292)	0.524
No	Reference		Reference	
**Housing status**				
Homeless	1.087 (0.385, 1.789)	**0.003**	0.844 (0.120, 1.568)	**0.022**
Unstable housing	0.425 (0.216, 0.634)	**<0.001**	0.326 (0.109, 0.543)	**0.003**
Stable housing	Reference		Reference	
**Mental health status**				
Yes	0.358 (0.122, 0.594)	**0.003**	0.309 (0.075, 0.544)	**0.010**
No	Reference		Reference	
**Viral load**				
Detectable	0.233 (−0.094, 0.560)	0.161	0.074 (−0.251, 0.398)	0.656
Undetectable	Reference		Reference	
**Total *N***			498	
**VIF**			<1.2	

CRD: crude risk difference, ARD: adjusted risk difference, CI: confidence interval, VIF: variance inflation factor; statistically significant *p* values are in bold.

## Data Availability

Underlying de-identified data and R code is available upon request from the corresponding author.

## Supplementary Materials

The following are available online at https://www.mdpi.com/article/10.3390/ijerph18158031/s1, Table S1: Association between individual characteristics and HIV stigma.
