# Supplementary material for: Psychosocial Determinants of HIV Stigma among Men Who Have Sex with Men in San Francisco, California
Source: Int J Environ Res Public Health. 2021 Jul 29;18(15):8031. doi: 10.3390/ijerph18158031 (PMC8345572; doi:10.3390/ijerph18158031)
Supplement: Supplementary file 1 [file ijerph-18-08031-s001.zip › Table S1.pdf]

Table S1. Association between individual characteristics and HIV stigma.

	Model 1		Model 2	
	**ARD (95% CI)**	***p*-value**	**ARD (95% CI)**	***p*-value**
**Age in years**				
13–29	0.241 (0.016, 0.466)	**0.036**		
30–49	0.217 (0.058, 0.377)	**0.008**		
50 and above	Reference			
**Race**				
Asian	0.009 (−0.281, 0.299)	0.953		
Black or African American	−0.005 (−0.263, 0.252)	0.967		
Hispanic or Latinx	−0.050 (−0.227, 0.126)	0.575		
Multiple	0.329 (0.060, 0.599)	**0.017**		
Other	0.343 (−0.081, 0.767)	0.113		
White	Reference			
**Injection history**				
Yes	0.171 (−0.038, 0.379)	0.108		
No	Reference			
**Housing status**				
Homeless			0.964 (0.254, 1.674)	**0.008**
Unstable Housing			0.416 (0.209, 0.622)	**< 0.001**
Stable housing			Reference	
**Mental health status**				
Yes			0.304 (0.076, 0.533)	**0.009**
No			Reference	
**Viral load**				
Detectable			0.117 (−0.190, 0.423)	0.455
Undetectable			Reference	
**Total *N***	541		538	
**VIF**	< 1.1		< 1.1	

ARD: adjusted risk difference, CI: confidence interval, VIF: variance inflation factor, significant *p* values are in bold..
